# The efficacy and safety of intravesical chondroitin sulphate solution in recurrent urinary tract infections

**DOI:** 10.1186/s12894-022-01149-7

**Published:** 2022-11-23

**Authors:** M. S. Rahnama’i, A. Javan Balegh Marand, K. Röschmann-Doose, L. Steffens, H. J. Arendsen

**Affiliations:** 1 St. Elizabeth- Tweesteden Hospital, Tilburg, The Netherlands; 2 Society of Urological Research and Education (SURE), Heerlen, The Netherlands; 3 Maastricht University Medical Center (MUMC+), Maastricht, The Netherlands; 4 G. Pohl-Boskamp GmbH & Co. KG, Hohenlockstedt, Germany; 5 Andros Clinics, Bladdercenter, The Hague, The Netherlands

**Keywords:** Recurrent urinary tract infections, Cystitis, Uropathogens, Intravesical instillation, Chondroitin sulphate, Antibiotic stewardship, Quality of life

## Abstract

**Background:**

Urinary tract infections are among the most common indications for antibiotic therapy. The emergence of resistant uropathogens indicates the need for treatment alternatives. Replenishment of the glycosaminoglycan layer of the bladder, achieved by intravesical instillation of e.g. chondroitin sulphate (CS), is described to be a cornerstone in the therapy of cystitis. To retrospectively evaluate the efficacy of a therapy with 0.2% CS in patients suffering recurrent urinary tract infections (rUTI) in comparison to a treatment with low-dose long-term antibiotics (LDLTAB) and a combination of both.

**Methods:**

A total of 151 patients with recurrent UTI who underwent intravesical therapy at Diaconesse hospital in Leiden, The Netherlands were included. 50 patients had been treated with CS, 51 patients had received LDLTAB, and 50 patients had received a combination therapy (LDLTABCS). Data recorded for baseline, after 6, and 12 months of treatment were evaluated. Descriptive statistics were calculated. Exploratory comparisons between groups and within groups were performed by using one-tailed and paired t-tests. Patients filled in a standardized quality of life questionnaire (EQ-5D).

**Results:**

We found a statistically significant reduction of number of infections from 7.10 ± 0.50 SEM to 0.45 ± 0.07 SEM after 12 months therapy with CS compared to 12 months therapy with LDLTAB (from 7.04 ± 0.47 SEM to 1.8 ± 0.15 SEM). The number of visits to the urologist significantly decreased in the CS group from 7.46 ± 0.80 SEM to 1.28 ± 0.11 SEM and from 4.10 ± 0.29 SEM to 1.35 ± 0.11 SEM in the LDLTABCS group. In addition, a significant increase in Quality of life (QoL) was seen in the CS-group (from 58.2 ± 0.82 SEM to 80.43 ± 0.82 SEM) and in the LDLTABCS group (from 62.4 ± 0.97 SEM to 76.73 ± 1.06 SEM). There was no improvement in QoL with LDLTAB (from 58.24 ± 1.08 SEM to 58.96 ± 1.19 SEM). Evaluation’s evidence is limited due to its retrospective character.

**Conclusions:**

Retrospective analysis of data from patients that underwent therapy for rUTIs confirms the safety and efficacy of CS and indicate a superiority to antibiotic treatment of rUTIs.

**Supplementary Information:**

The online version contains supplementary material available at 10.1186/s12894-022-01149-7.

## Introduction

Typical symptoms of chronic and recurrent cystitis are dysuria, imperative urinary urgency, increased urinary frequency, and suprapubic pain. Affected patients suffer to a considerable degree from a massive decrease in quality of life (QoL), induced by the increased urinary frequency, leading to social impairment [1–3].

Approximately 20% to 30% of women suffer from recurrent urinary tract infections (rUTIs), defined as two or more culture-proven UTIs in 6 months or three or more UTIs in one year [4, 5]. Although the etiology of UTIs is mainly bacterial and uropathogenic *E. coli* (UPEC) is the most common pathogen, antibiotic therapy does not reach a significant rate of success in preventing recurrent episodes [6]. As rUTIs likely result from the dysbiosis of gut microbiome leading to a persistent reservoir of UPEC in the intestine thus antibiotic treatment cannot attenuate but potentially facilitates the underlying cause [7].

Another potential cause of recurrent episodes is bacterial internalization and development of intracellular bacterial communities [8]. It is believed that bacterial attachment but also internalization is favoured by a damaged urothelial mucous membrane [8–10].

Furthermore, repeated antibiotic treatment increases the risk for developing drug resistance [11]. As shown within a long-term surveillance study the activity in terms of clearing the infection for (among others) Fosfomycin was found to decrease significantly over the 7-year study period [12] although much lower frequencies of resistance were observed in most European countries as well [13]. Given the alarming emergence of resistant uropathogens and the global increase in resistant strains [14, 15], sustainable treatment alternatives will support antibiotic stewardship.

A key element in the pathogenesis of chronic forms of cystitis is the glycosaminoglycan (GAG) layer, which regulates the penetration of solutes from within the urine to the bladder wall. The physiological function of this layer is to protect the urothelium. Endogenously produced chondroitin sulphate (CS) has been shown to be the dominant sulphated GAG on the urothelial luminal surface [9]. Many forms of chronic cystitis indicate a deficit in key elements of the GAG layer [16–19], which is regarded as an important element in the development of various diseases. A defect of the GAG layer promotes a chronically recurring infection as the protection from uropathogenic bacteria is reduced [18–21]. The replenishment of the GAG layer is described to be a cornerstone in the therapy of cystitis and the effectiveness of CS for treatment in chronic cystitis has been demonstrated [22–24]. CS with a concentration of 0.2% is in favour for the intravesical GAG layer replenishment therapy [25].

Gepan® instill is a certified and marketed class III medical device. It contains 0.2% sodium chondroitin sulphate (CS) as its physically effective component, and is instilled intravesically into the bladder and restores or reinforces the protective function of the GAG layer [26, 27].

The objective of this retrospective study was to evaluate the efficacy and safety of a therapy with 0.2% CS in patients suffering rUTIs in comparison to low-dose long-term antibiotics (LDLTAB).

## Material and methods

### Patients

Data retrospectively derived from 151 patients that underwent therapy for the treatment of rUTIs at Diaconessen Hospital Leiden (The Netherlands) were analyzed. Patients were chronologically selected from the hospital’s database based on start of treatment and the selection process was stopped, as soon as 50 patients per group were identified that met all in- and none of the exclusion criteria. 50 patients had been treated with 0.2% CS (CS), 50 patients with a combination of CS and low-dose long-term antibiotics (LDLTABCS), and 51 patients had received low-dose long-term antibiotics (LDLTAB). According to EAU guidelines [28], inclusion criteria focused on patients that experienced at least 3 episodes of uncomplicated cystitis in the last 12 months prior to the start of treatment, or 2 episodes in the last 6 months prior to the start of treatment as well as a positive culture of uropathogens at ≥ 10^3^ CFU/ml. Data from patients younger than 18 years, pregnant patients, patients with a positive urine culture of uropathogens ≤ 10^3^ CFU/ml, patients with urogenital defects/abnormalities, or a post voiding residual volume of > 50 ml were excluded from this retrospective data analysis. Medical histories of the patients were documented prior to the start of treatment with CS and/or LDLTAB. Data recorded for baseline, after 6 months, and 12 months of treatment were evaluated for efficacy and safety of treatment.

### Study design and treatment regime

Within hospital’s routine patients were asked, whether they would agree to evaluation and publication of data obtained during their treatment for research purpose and gave informed consent electronically. This retrospective analysis has been approved by the Ethics Committee at Diaconessen Hospital Leiden, in accordance with the provisions of the Declaration of Helsinki. Data of patients were not included within this analysis, if the in- and exclusion criteria were not met.

Prior to the start of treatment, the medical history of the past 12 month was documented. Additionally, the parameters defined were documented prior to start of the treatment, after 6 months, and after 12 months of treatment.

Patients received either CS and/or LDLTAB. According to the instructions for use and the treatment regime established at Diaconessen Hospital Leiden, patients receiving CS or LDLTABCS were treated with 40 ml of 0.2% CS solution (Gepan® instill) per instillation. The treatment was applied by a trained nurse under the supervision of the attending urologist, or by the urologist (see Additional file 1 for further details on the protocol for instillation). Following the first instillation, patients underwent the following protocol: weekly instillations for a duration of 6 weeks, followed by instillations every 2 weeks for 2 months, which were followed by instillations every 3 weeks for approximately 2 months, with subsequent instillations every 6 weeks up to a total duration of 1 year. For treatment with LDLTAB alone or in combination with CS patients received one of the following antibiotics decided by the physician in charge based on the treatment regimens valid at the hospital in accordance with respective treatment guidelines and based on the patient’s individual characteristics and anamnesis on the individual antibiotic treatment: Furadantine® (Nitrofurantoin, 50 mg, 1 × day, 6 months), Noroxin® (Norfloxacin, 400 mg, 2 × day, 3 months), Augmentin® (Amoxicillin/clavulanic acid, 625 mg, 1 × day, 3 months), Monotrim® (Trimethoprim, 100 mg, 1 × day, 6 months) or Ciproxin® (Ciprofloxacin, 500 mg 1 × day, 3 months). Prior to the start of treatment, after 6 months of treatment, and after 12 months of treatment, patients filled in a standardized quality of life questionnaire (EQ-5D) according to the hospitals’s treatment routine.

### Study parameters

Patient number, date of birth, gender and body mass index were noted to demographically describe the patients. The following documented parameters have been collected and evaluated: date of visits/start of treatment, confirmed dates of infections, positive urine culture for uropathogens (≥ 10^3^ CFU/ml), identification of uropathogens, antibiotic sensitivity, prescribed antibiotic treatment, cystoscopy, QoL (EQ-5D), visits to the specialist.

### Data management and evaluation

Pseudonymized data were obtained from Diaconessen Hospital Leiden. Patient identities were substituted by an identification code that was recorded on any form submitted to the sponsor of this data evaluation.

Data entry, check for quality and plausibility (double-checked each) and evaluation took place from March to July 2015. The following descriptive statistics were calculated: N (number of non-missing observations), mean (arithmetic mean), standard deviation (SD), standard error of the mean (SEM). For categorical data (nominal and ordinal variables) absolute and relative frequencies were calculated. Exploratory comparisons between groups and within groups were performed by using one-tailed and paired or unpaired t-tests.

The determination of the sample size was not based on biometric considerations. However, the sample size was considered to be sufficient to meet the objectives of this retrospective study.

## Results

### Demographic characteristics

Median age, BMI, and distribution of gender were comparable among the groups. The proportion of female patients within all groups investigated was high, reflecting the disease’s target population (Additional file 1: Table S1).

### Assessment of confirmed infections

Infections were confirmed by positive urine culture. In the majority of patients, *E. coli* was the most prominent uropathogen, with a minimum prevalence of 86% prior to the start of treatment. Other detected uropathogens were *E. faecalis*, *Citrobacter*, *P. mirabilis* and *Klebsiella* which were only of minor importance and prevalence declined even further to single occurrences at later stages of treatment (Additional file 1: Table S2). All uropathogens detected at baseline were tested and with exception of one case in the LDLTABCS group were found to be susceptible to treatment with AB. At 6 months follow-up 4 cases of detected *E. coli* in the LDLTABCS group were evaluated not to be susceptible to AB treatment.

**Fig. 1:**
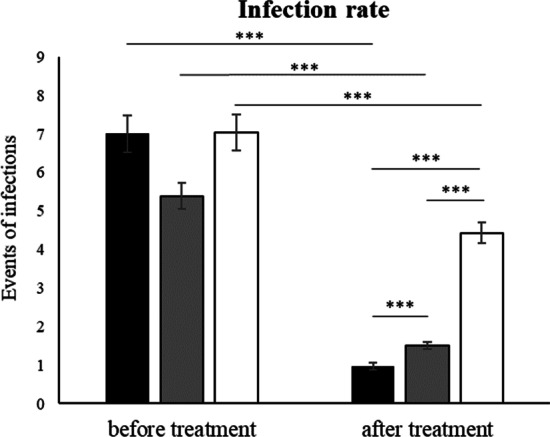
Assessment of infection rates expressed as number of infections in rUTI patients 12 months before treatment and after 12 months of treatment. Illustrated are mean events of infections per group; CS (n = 50, black bars), LDLTABCS (n = 50, grey bars), LDLTAB (n = 51, white bars); ***p < 0.00001

Patients who received CS experienced 7.10 infection episodes (SEM ± 0.50) on average within 12 months prior to the start of treatment. After 6 months, number of infections showed a significant decline to 0.55 infections (SEM ± 0.08), and an additional reduction to 0.45 infections (SEM ± 0.07) after 12 months of treatment. Patients with LDLTABCS were initially assessed to have experienced 5.38 UTIs (SEM ± 0.34) within 12 months before the start of the treatment. After 6 months this number declined to 1.24 infections (SEM ± 0.08), and after 12 months the number of infections showed a further reduction to 0.26 infections (SEM ± 0.06). Patients that received LDLTAB experienced on average 7.04 infections (SEM ± 0.47) within 12 months prior to the start of the treatment. After 6 months, this number showed a reduction to 2.67 UTIs (SEM ± 0.14), with a further decrease to 1.8 infections (SEM ± 0.15) after 12 months. As shown in Fig. 1, all treatments resulted in a significant decrease in the number of infections when periods before treatment were compared to the number of infections that occurred during the treatment period of 12 months. Reduction in infection rate as induced by CS and LDLTABCS was found to be significant when compared to LDLTAB. Furthermore, the number of infections showed a significant reduction with CS after 12 months of treatment, when compared to LDLTABCS.

### Assessment of quality of life

**Fig. 2:**
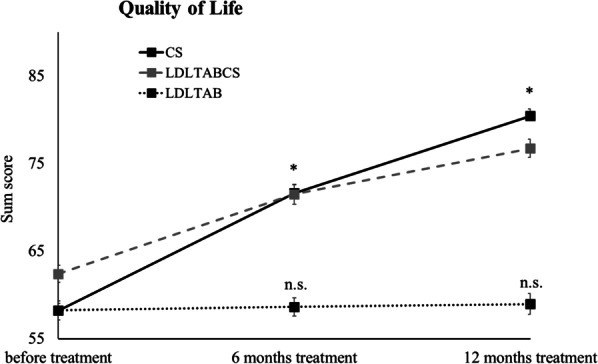
Assessment of quality of life. Shown are mean QoL scores per group; CS (n = 50, black line), LDLTABCS (n = 50, grey line), LDLTAB (n = 51, black and dotted line); *p < 0.00001; n.s. not significant, p > 0.05

Patients’ QoL was assessed in a standardized quality of life questionnaire (QoL; EQ-5D) prior to treatment and after 6 and 12 months of treatment. On a scale ranging from 0 to 100 patients that received CS had a score of 58.2 (SEM ± 0.82) before the start of treatment. After 6 and 12 months this score significantly increased to 71.60 (SEM ± 0.98) and 80.43 (SEM ± 0.82), respectively. Patients’ QoL scores in the LDLTABCS group initially showed a score of 62.4 (SEM ± 0.97), increasing to 71.5 (SEM ± 1.13) after 6 months, and 76.73 (SEM ± 1.06) after 12 months of treatment. The initial QoL score of patients that received LDLTAB alone was 58.24 (SEM ± 1.08). After 6 and 12 months of treatment the QoL scores in this group were 58.63 (SEM ± 1.04), and 58.96 (SEM ± 1.19), respectively, indicating no significant improvement (see Fig. 2).

### Quantification of uropathogens

**Fig. 3:**
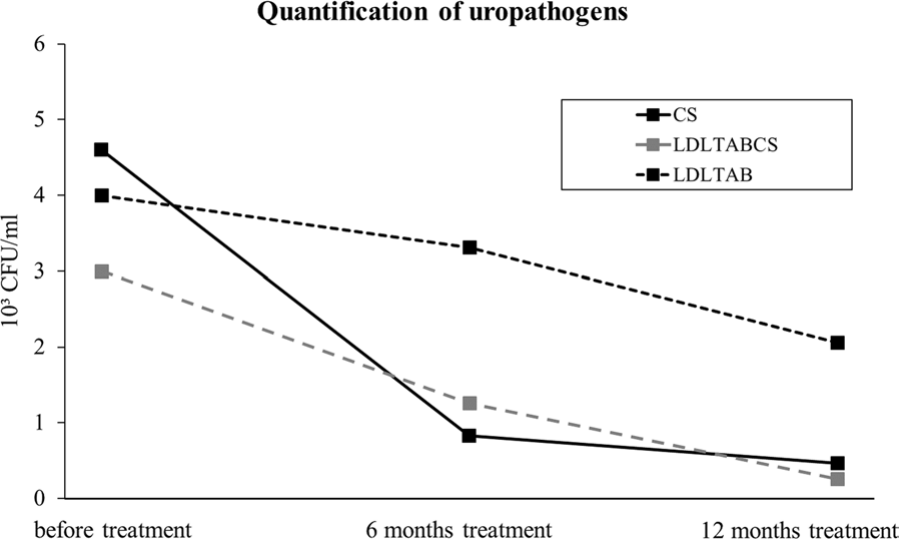
Quantification of uropathogens in urine cultures from rUTI patients. Values are given as mean number × 10^3^ colony forming units (CFU)/ml per group; CS (n = 50, black line), LTLDABCS (n = 50, grey line), LDLTAB (n = 51, black dotted line)

Prior to the start of treatment, after 6 months and after 12 months of treatment uropathogens were quantified from urine culture (Fig. 3), using standard microbiological methods. The urine cultures of the patients with CS initially showed an uropathogen load of 4.60 × 10^3^ CFU/ml (SEM ± 0.29), which was markedly reduced to 0.83 × 10^3^ CFU/ml (SEM ± 0.09) after 6 months, and 0.47 × 10^3^ CFU/ml (SEM ± 0.08) after 12 months of treatment. Patients with LDLTABCS initially displayed an uropathogen load of 3.00 × 10^3^ CFU/ml (SEM ± 0.16), with a clear decrease in bacterial load after 6 months with 1.26 × 10^3^ CFU/ml (SEM ± 0.09) and 12 months with 0.26 × 10^3^ CFU/ml (SEM ± 0.07), respectively. Patients with LDLTAB showed an initial titer of 4.00 × 10^3^ CFU/ml (SEM ± 0.17), which only slightly decreased to 3.31 × 10^3^ CFU/ml (SEM ± 0.20) after 6 months. After 12 months of treatment with LDLTAB, urine cultures still contained a substantial uropathogen load of 2.06 × 10^3^ CFU/ml (SEM ± 0.18).

### Assessment of visits at the urologist

Recurrent UTIs-induced visits to the urologist completed by patients within 12 months prior to the start of the treatment and during treatment were recorded and evaluated. Before the start of therapy patients treated with CS completed 7.46 (SEM ± 0.80) visits to the urologist. After 6 months the mean number of visits was reduced to 1.17 (SEM ± 0.10) and did not change remarkably after 12 months of treatment (1.28; SEM ± 0.11). Patients that received LDLTABCS completed on average 4.10 (SEM ± 0.29) visits within 12 months prior to the start of the treatment. After 6 months the frequency of visits showed a notable reduction to 1.78 (SEM ± 0.10) visits, with a further decrease to 1.35 (SEM ± 0.11) visits after 12 months of treatment. As the number of visits to the specialist 12 months prior to the start of treatment was not available for patients receiving LDLTAB, only data obtained for 6 and 12 months of treatment could be evaluated. After 6 months of treatment, this group of patients on average completed 3.00 (SEM ± 0.17) visits at the urologist. This frequency showed a moderate reduction to 2.04 (SEM ± 0.17) visits after 12 months of treatment (data not shown). Treatment with CS alone or in combination with LDLTAB induced a significant decrease in doctor’s visits, whereby CS treatment resulted in a more pronounced decrease, with a significant reduction at the end of treatment when compared to the LDLTABCS group (Additional file 1: Fig. S1).

### Safety

As this study was designed as retrospective analysis and only data from patients undergoing the complete therapy were included, no terminations of treatment due to the occurrence of any adverse event were recorded. However, for three patients that received CS mild to moderate burning feeling when urinating was documented after catheterization. Although no causality to CS was evaluated according to physician’s assessment, induction of these adverse events via the procedure of catheterization is possible. No adverse events were reported from patients receiving antibiotics.

## Discussion

The aim of this study was to evaluate the efficacy of a treatment for rUTIs with either CS, LDLTAB or the combination LDLTABCS. The majority of patients were female (94% to 96%), reflecting the known prevalence for this disease [15]. The prevalence of *E.coli*, as confirmed by positive urine culture, was found to range from 86 to 100%, which is in line with data from literature where *E.coli* is described to be the predominant uropathogen with prevalence’s of 75–90% [5, 10].

During the course of the therapy with CS, the number of confirmed infections in patients was significantly reduced. In comparison with the LDLTAB group this decline was more pronounced in the CS group. Moreover, patients did not benefit from a combination of CS and LDLTAB, as QoL and visits at the urologist were comparable to the group of patients that received CS alone. Interestingly, CS instillation alone induced a significantly higher decrease in infection rate after 6 months when compared to the combination. Given the fact that the procedure of instillation bears additional risks for the induction of catheter-associated UTIs [29], the massive decrease of infection rates observed within the groups of patients that received CS bladder instillations might be even more substantial.

Compared to treatment with LDLTAB, urine cultures from patients that received CS clearly showed a more reduced uropathogenic load throughout the treatment period. Corresponding to the decrease in a number of infections and the identified uropathogenic load, treatment with CS also more distinctly reduced the number of specialist visits, when compared to patients that received LDLTAB.

Several studies have demonstrated that therapies utilizing intravesical instillations with hyaluronic acid and/or CS to replenish the GAG layer are more effective in reducing rUTIs than therapies with long-term antibiotics [6, 30–34]. In line with the findings of the present retrospective analysis, a combination of these therapies with LDLTAB failed to further contribute to the efficacy of treatment [6]. Given the worldwide spread of resistant strains, down-regulation of the use of antibiotics should be a primary aim. In addition to the increasing risk of resistant infections, this may also pose an additional risk of promoting the expression of virulence factors [35]. In this study, in total five cases of antibiotic resistance were observed corresponding to a frequency of 3% which matched the typically expected frequency [13, 36]. There clearly is a need to reevaluate the effectiveness of prophylactic strategies in patients with highly rUTIs, especially during short-term treatments, or when individuals are non-compliant. Besides the efficacy parameters that clearly indicate a superiority of CS bladder instillations in comparison to antibiotics, patients treated with CS also experienced a more pronounced increase in QoL, whereby patients < 60 years reached significantly higher QoL scores than patients ≥ 60 years (Data not shown).

As with all retrospective analyses of clinical data an underestimation of the occurrence of adverse events and in turn, an overestimation of the efficacy has to be considered, as only data from a patient that did not terminate the therapy were included in the evaluation.

Because of the retrospective character of this study we have no information about additional alternative treatments e.g. Cranberry or D-Mannose.

However, this evaluation clearly demonstrates the benefits of GAG replenishment as a treatment for rUTIs. This is in line with recent recommendations of the EAU for the treatment of rUTIs in adult women, where non-antimicrobial prophylaxis (e.g. endovesical instillations) is given priority over the use of antibiotics [37]. A recent study showed lasting clinical benefits for up to 36 months after end of treatment [38]. However, well-designed large-scale clinical studies are needed to confirm these results and to provide solid evidence for guideline recommendations.

## Supplementary Information

**Additional file 1**. Methods: Protocol of instillations and antibiotic treatment. Table S1: Baseline patient characteristics (n = 50/51 per group). Table S2: Prevalence of bacterial pathogens in confirmed UTIs. Figure S1: Number of rUTI-induced visits to the urologist 12 months before and after treatment. Shown are mean values for number of visits per group; CS (n = 50, black bars), LDLTABCS (n = 50, grey bars); ** p = 0.009; *** p < 0.00001.

## Data Availability

The datasets generated and/or analysed during the current study are available from the corresponding author on reasonable request.

## Abbreviations

CS: Chondroitin sulphate
GAG: Glycosaminoglycan
LDLTAB: Low-dose long term antibiotics
LDLTABCS: Low-dose long term antibiotics + chondroitin sulphate
QoL: Quality of life
rUTI: Recurrent urinary tract infection
SD: Standard deviation
SEM: Standard error of the mean
UPEC: Uropathogenic *E. coli*
