# Supplementary material for: The efficacy and safety of intravesical chondroitin sulphate solution in recurrent urinary tract infections
Source: BMC Urol. 2022 Nov 23;22:188. doi: 10.1186/s12894-022-01149-7 (PMC9685912; doi:10.1186/s12894-022-01149-7)
Supplement: Supplementary file 1 — Additional file 1. Methods: Protocol of instillations and antibiotic treatment. Table S1: Baseline patient characteristics (n = 50/51 per group). Table S2: Prevalence of bacterial pathogens in confirmed UTIs. Figure S1: Number of rUTI-induced visits to the urologist 12 months before and after treatment. Shown are mean values for number of visits per group; CS (n = 50, black bars), LDLTABCS (n = 50, grey bars); ** p = 0.009; *** p < 0.00001. [file 12894_2022_1149_MOESM1_ESM.docx]

**Supplementary Material**

**The efficacy and safety of intravesical chondroitin sulphate solution in recurrent urinary tract infections**

M.S. Rahnama’i^1and 2^, A. Javan Balegh Marand^2 and 3^, K. Röschmann-Doose^4^, L. Steffens^4^ and H.J. Arendsen^5^

**Affiliations**

^1^ St. Elizabeth- Tweesteden Hospital in Tilburg, The Netherlands

^2^ Society of Urological Research and Education (SURE), Heerlen, The Netherlands

^3^ Maastricht University Medical Center (MUMC+), The Netherlands

^4^ G. Pohl-Boskamp GmbH & Co. KG, Hohenlockstedt, Germany

^5^Andros Clinics, Bladdercenter, The Hague, The Netherlands

**Corresponding author**

**L. Steffens**

**l.steffens@pohl-boskamp.de**

**Supplemental Methods:**

**Protocol of instillations and antibiotic treatment**

Patient received 40 ml of 0.2% CS solution (Gepan^®^ instill) per instillation whereby the instillation was performed according to the instructions for use. The treatment was applied by a trained nurse under the supervision of the attending urologist, or by the urologist. The entire volume of Gepan^®^ instill should be warmed up to room or body temperature and was instilled by means of catheder after the bladder had been completely emptied. Gepan^®^ instill should have remained in the bladder as long as possible, at any rate for a minimum of 30 minutes.

Following the first instillation, patients underwent the following protocol: weekly instillations for a duration of 6 weeks, followed by instillations every 2 weeks for 2 months, which were followed by instillations every 3 weeks for approximately 2 months, with subsequent instillations every 6 weeks up to a total duration of 1 year.

Treatment with LDLTAB alone or in combination with CS followed the same schedule as the CS treatment and patients received one of the following antibiotics: Furadantine® (Nitrofurantoin, 50 mg, 1 x day), Noroxin® (Norfloxaxin, 400 mg, 2 x day), Augmentin® (Amoxicillin/clavulanic acid, 625 mg, 1 x day), or Monotrim® (Trimethoprim 100 mg, 1 x day) or Ciproxin® (Ciprofloxacin, 500 mg 1 x day, 3 months) .

**Supplement Table S1:** Baseline patient characteristics (n = 50/51 per group)

| Baseline characteristic (CS) | Value |
| --- | --- |
| Median age (years; min/max) | 61.1 (23.8/84.8) |
| Gender |  |
| Female (n/relative) | 48 (96) |
| Male (n/relative) | 2 (4) |
| Median BMI (min/max) | 28.0 (20.7/42.0) |
| Partner (n/relative) | 40 (80) |
| Sexually Active (n/relative) | 34 (68) |
| Baseline characteristic (LDLTABCS) | **Value** |
| Median age (years; min/max) | 64.8 (18.6/93.0) |
| Gender |  |
| Female (n/relative) | 47 (94) |
| Male (n/relative) | 3 (6) |
| Median BMI (min/max) | 29.0 (20.1/41.3) |
| Partner (n/relative) | 46 (92) |
| Sexually Active (n/relative) | 35 (70) |
| Baseline characteristic (LDLTAB) | **Value** |
| Median age (years; min/max) | 60.0 (21.7/91.9) |
| Gender |  |
| Female (n/relative) | 48 (94) |
| Male (n/relative) | 3 (6) |
| Median BMI (min/max) | 30.0 (21.0/44.2) |
| Partner (n/relative) | 45 (88) |
| Sexually Active (n/relative) | 35 (70) |

**Supplement Table S2:** Prevalence of bacterial pathogens in confirmed UTIs

|  | *E. coli* | *Citrobacter* | *E. faecalis* | *P. mirabilis* | *Klebsiella* |
| --- | --- | --- | --- | --- | --- |
| CS |  |  |  |  |  |
| Before treatment | 86% | 4% | 2% | 6% | 2% |
| 6 months of treatment | 88% | 8% | - | 4% | - |
| 12 months of treatment | 95% | - | 5% | - | - |
| LDLTABCS |  |  |  |  |  |
| Before treatment | 96% | 4% | - | - | - |
| 6 months of treatment | 100% | - | - | - | - |
| 12 months of treatment | 100% | - | - | - | - |
| LDLTAB |  |  |  |  |  |
| Before treatment | 98% | 2 | - | - | - |
| 6 months of treatment | 94% | 4% | 2% | - | - |
| 12 months of treatment | 95% | 5% | - | - | - |


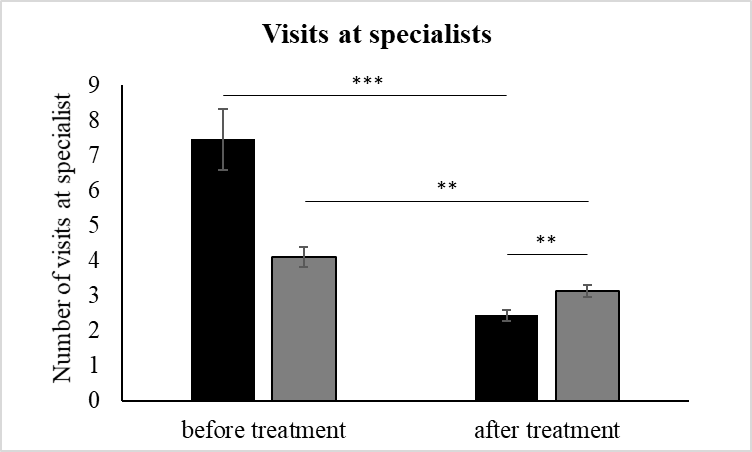


**Supplemental Figure S1:** Number of rUTI-induced visits to the urologist 12 months before and after treatment. Shown are mean values for number of visits per group; CS (n = 50, black bars), LDLTABCS (n = 50, grey bars); ** p = 0.009; *** p < 0.00001.
